# Organic waste compost and spent mushroom compost as potential growing media components for the sustainable production of microgreens

**DOI:** 10.3389/fpls.2023.1229157

**Published:** 2023-07-04

**Authors:** Pradip Poudel, Anela E. K. Duenas, Francesco Di Gioia

**Affiliations:** ^1^ Department of Plant Science, The Pennsylvania State University, University Park, PA, United States; ^2^ College of Natural and Applied Sciences, University of Guam, Mangilao, GU, United States

**Keywords:** alternative substrate, coconut coir, peas, peat, peat-substitute, nutrient recycling, radish

## Abstract

Microgreens are emerging specialty crops becoming increasingly popular for their rich nutrient profile and variety of colors, flavors, and textures. The growing medium is a significant key factor in microgreen yield, quality, and sustainability. The widespread use of peat-based media raises questions regarding the environmental sustainability of microgreens production, and new substrates that are more sustainable are required. To this purpose, a study was designed with the objective of comparing eight alternative growing media evaluating their physicochemical properties and effect on yield, mineral profile, and nutritional quality of peas and radish microgreens. Tested substrates included a standard peat and perlite mixture (PP), coconut coir (CC), spent mushroom compost (SMC), organic waste compost (CMP), and 50:50 (v:v) mixes of PP and SMC, PP and CMP, CC and SMC, and CC and CMP. The physicochemical properties widely differed among the alternative substrates tested. SMC had high electrical conductivity and salt concentration, which resulted in poor seed germination. Growing media tested significantly influenced the production and nutritional quality of both microgreen species and variations were modulated by the species. With a 39.8% fresh yield increase or a small yield decrease (-14.9%) in radish and peas, respectively, PP+CMP (50:50, v/v) mix provided microgreens of similar or higher nutritional quality than PP, suggesting the potential of substituting at least in part peat with CMP. Using locally available CMP in mix with PP could reduce the microgreens industry reliance on peat while reducing costs and improving the sustainability of the production of microgreens. Further research is needed to evaluate also the potential economic and environmental benefits of using locally available organic materials like CMP as alternative growing media and peat-substitute to produce microgreens.

## 1 Introduction

Microgreens are emerging specialty crops increasingly popular among chefs and consumers for their aesthetic properties and rich nutritional profile (Kyriacou et al., 2016; Di Gioia et al., 2021). Often assimilated to sprouts, microgreens differ from sprouts in the production process, harvesting stage, and what constitutes the edible portion (Di Gioia et al., 2017b). Grown from a variety of edible seeds, the edible portion of microgreens is represented by their young shoots, harvested at an early stage of development, when the cotyledons are fully expanded, or the first true leaves have just formed. Unlike sprouts, microgreens do not include the radicles or seed casing in the edible portion (Di Gioia et al., 2017b). From a production standpoint, while sprouts are generally produced without a substrate, using only recirculating water, and mainly in the absence of light, microgreens are produced in the presence of sunlight or artificial lighting and generally require a growing medium. Moreover, having a longer growth cycle, microgreens benefit more than sprouts from the availability of nutrients either through the substrate or through the supply of a nutrient solution. Yield performance and the nutritional value of microgreens largely differ based on the species, management practices, and growing environment (Kyriacou et al., 2016; Mishra et al., 2022). Besides the availability of high-quality seeds, one of the major inputs required to produce microgreens and determining the sustainability of the production process is the GM used (Di Gioia et al., 2017a; Di Gioia et al. 2017b; Li et al., 2021). Peat and peat-based media are the most commonly used GM for microgreens production as they have optimal physicochemical properties, are widely available, and generally provide sufficient nutrition for the production of microgreens (Kyriacou et al., 2016). However, there is increasing concern about the sustainability of peat-based media, as peat is considered a non-renewable resource and its harvest is a primary source of carbon emissions (Turetsky et al., 2015).

Reducing the use of peat-based GM could improve the sustainability of microgreens production, reducing carbon emissions from peat land. Additionally, peat-moss price has increased in recent years due to shortages caused by supply chain issues and to the strict regulation or ban of the commercial peat harvest in some of the countries producing peat, like Canada and Ireland (Roach, 2022). In this context, there is a big need to find more sustainable alternatives to peat-based substrates and reduce peat use, at least in part (Di Gioia et al., 2017a).

Besides ensuring the achievement of good yield and quality performances, peat-substitute GM should be available in large amounts, at a relatively low cost, and should be environmentally sustainable (Di Gioia et al., 2017a; Gruda, 2019). A sustainable alternative to peat-based media, commonly available at commercial level, is coconut coir, a by-product of the coconut industry, renewable, and of organic origin. However, it is unlikely that a single material like coconut coir could fully substitute peat providing similar crop yield and quality performances. In fact, peat itself is often mixed with other materials to obtain substrate mixes with optimal physicochemical properties. Instead, most likely, peat could be replaced or partially substituted by a substrate mix. In this perspective, there is a great opportunity to reduce production costs and improve the environmental sustainability of the production of microgreens by using locally available organic resources and by-products that may be recycled as peat-substitute in substrate mixes. Combining peat or coconut coir with organic waste composts could reduce the consumption of pea-based media while contributing to recycle organic waste and by-products generated locally. Organic waste composts are commonly generated and could be recycled as a component of GM at the household level, at municipal level, or at a larger scale. Similarly, by-products of the agri-food industry are often available and could be recycled at a regional level (Petropoulos et al., 2019b). For instance, the mushroom industry produces abundant spent mushroom compost (SMC), with Pennsylvania alone producing around 1.1 million tons of SMC annually. Due to Pennsylvania’s leading position in the mushroom industry in the United States, which accounts for approximately 62.9% of total mushroom volume sales, SMC recycling as a component of growth medium has considerable promise in this region (USDA, 2022). Prior to removing the spent substrate from the mushroom house, growers “pasteurize” it with steam to eliminate any pests and diseases that may be present in the substrate and casing (Beyer, 2011). Currently, after being steamed SMC is either composted or used as a soil amendment. Spent mushroom compost has about 25% organic matter content, has a neutral or close to neutral pH, and a good level of macro and micronutrients; however, in some cases, it may contain high soluble salt levels (Fidanza et al., 2010), which may not be ideal for growing most microgreen species. Mixing SMC with other GM could dilute the soluble salts if present in high concentration, making it potentially suitable as an alternative medium for microgreen production. Taking advantage of organic resources available locally and containing nutrients could substantially reduce the cost of production of microgreens and improve the sustainability of their production process, while contributing to reduce the consumption of peat and recycling organic material that otherwise would be considered a waste or a soil amendment in the best-case scenario. The opportunity to use resources locally available to produce microgreens at relatively low cost may also facilitate the microscale production of microgreens for self-consumption at the household level, which may potentially contribute to address nutrition security issues in food deserts and regions affected by malnutrition (Di Gioia et al., 2021; Poudel et al., 2023). On the other hand, at commercial level, microgreens are considered high value fresh products for which both the aesthetic and intrinsic nutritional quality are key, and it is critical to ensure that alternative substrate material do not have a negative impact on yield and quality.

Therefore, a study was designed to compare coconut coir, organic waste compost, SMC, and their mixtures with a commercial peat-perlite mix commonly used for microgreen production. The alternative GM mixes tested were examined for their chemical properties and mineral concentration and the impact on microgreens’ yield and nutritional quality using pea and radish microgreens as test crops.

## 2 Material and methods

### 2.1 Experimental site, treatments, and experimental design

The study was conducted under controlled environmental conditions in the greenhouse facility of the Pennsylvania State University at University Park, PA.

Eight alternative growing substrates were tested to produce ‘Dwarf grey sugar’ peas (*Pisum sativum* L.) and ‘Red Rambo’ radish (*Raphanus sativus* L.) microgreens. The alternative GM tested were: 1) a standard peat and perlite mix (Sunshine Mix 4, Sun Gro Horticulture, Agawam, MA, USA), 2) coconut coir (Fiber Dust, Glastonbury, CT, USA), 3) spent mushroom compost (SMC) obtained from the Penn State Mushroom Research Center, 4) organic waste compost (CMP) obtained from the Penn State Composting Facility (PSU compost), 5) peat-perlite + SMC mix (50:50, v:v), 6) peat-perlite + compost mix (50:50, v:v), 7) coconut coir + SMC mix (50:50, v:v), and 8) coconut coir + compost mix (50:50, v:v). Each growing substrate was used to fill 12 cm × 16 cm × 5 cm black growing trays with drainage holes at the bottom, and representative samples of each growing medium were collected to analyze their physicochemical properties. Compressed coconut coir was rehydrated in deionized water according to manufacturer instructions before being used to prepare the substrate mixes, fill growing trays, and before collecting a sample for analysis. Treatments were arranged according to a completely randomized split-plot experimental design with three replications. Substrates were randomized in the main plots, and the two species were randomized in subplots. There were 8 large trays (1 per growing medium), each containing 6 small trays (12 cm × 16 cm × 5 cm, 3 per species) per replication. Each experimental unit included three small growing trays (sub-plot), and the trays of the two species were set in larger (25.4 cm × 50.8 cm) trays (main plot) with draining holes at the bottom.

### 2.2 Plant material, seed sowing, growing conditions, and microgreen harvest

Pea and radish seeds of high quality specifically marked to produce microgreens were purchased from Johnny’s Selected Seeds (Winslow, Maine, USA) and had a germination rate of 96% and 98%, and 7.3 and 97 seeds per g, respectively. Pea seeds were soaked in deionized water overnight before sowing, while radish seeds were directly seeded. The small growing trays of each substrate were seeded with 27.4 g of pea seeds and 4 g of radish seeds, establishing a seed density of 1 and 2 seeds/cm^2^, respectively. The seed density for the two species was defined based on previous studies considering seeds size and average shoot size of the two species. All the growing trays filled with the alternative GM tested were watered to saturation *via* sub-irrigation and let drain on a growing bench before seeding. After seeding, a weight was placed on top of the growing trays to press and hold the seeds in place, and a black polyethylene film was used to create a dark environment during germination. After the seeds germinated, weights and black covers were removed. Growing trays were misted daily from the top for the first two days until cotyledon formation, after which the trays of each growing medium were irrigated *via* sub-irrigation using tap water. Minimum and maximum greenhouse temperatures were set at 23.8 and 26.6°C, respectively. During the microgreen growing period, the average greenhouse temperature was 25.3°C.

Between 6:00 a.m. and 8:00 p.m., supplemental LED lighting (Illumitex ES24812 Eclipse Surexi Double Bar LED Grow Lights) was automatically turned on daily when solar radiation was below 1,000 w/m^2^. Radish and pea microgreens were harvested 5 days after sowing in presence of fully expanded cotyledons before the first true leaf formation.

Microgreens were harvested from each growing tray by cutting shoots right above the substrate using clean scissors. The fresh weight of microgreens harvested from each tray was measured on a laboratory scale, and a subsample of 20 shoots randomly selected from the middle of each growing tray was weighed to estimate the mean fresh weight of single shoots. As three small growing trays were available for each growing medium per species combination, the microgreens harvested from one small tray were oven dried at 65°C to measure dry weight. Oven-dried samples were milled by passing through a 1-mm sieve and used to analyze the mineral profile. Microgreens harvested from the other two small growing trays were stored at -80°C, freeze-dried, and used for phytochemical analysis after grinding.

### 2.3 Growing media physicochemical properties analysis

Two representative subsamples were collected from all studied GM and immediately submitted to the Penn State Agricultural Analytical Services Laboratory to analyze each substrate’s main chemical properties. The saturated media extract method with diethylenetriaminepentaacetic acid (DTPA) was used for the sample extraction, and each extracted sample was analyzed by measuring pH, electrical conductivity (EC), nitrate-nitrogen, ammonium-nitrogen, P, K, Ca, Mg, B, Cu, Fe, Mn, Na, Zn, and sulfate using the methods described in Warncke (1995). Organic matter (% on a dry weight basis) was calculated using the weight loss on ignition methods according to Schulte and Hoskins (1995).

Physical properties of GM were analyzed using the method described in Niedziela and Nelson (1992) and Di Gioia et al., 2017a with slight modification. Three representative subsamples were collected filling the core sample cylinders, whose bottom was covered with cheesecloth held in place by a rubber band. Growing media were saturated slowly by capillarity dipping the bottom of each core sample cylinder in water for 24 h and letting them drain overnight under gravitational force. Growing media within a core sampler were oven-dried at 65°C for a week until constant weight. The following parameters were measured and calculated during the above-described process; media volume (MV), saturated weight of the media (SWM), drain water volume (DWV), dry weight of the media (DWM). The following formulas were used to calculate the bulk density (BD), particle density (PD), water holding capacity (WHC), total pore space (TPS), and air-filled porosity (AFP). All the weights used in the following equations are expressed in g, and volumes are expressed in cm^3^.


Eq (1) $$\text{BD (}g/cm^{3}\text{)=}\frac{DWM}{MV}$$

Eq (2) $$\text{PD (}g/cm^{3}\text{)=}\frac{DWM}{MV-DWV-(SWM-DWM)}$$

Eq (3) $$\text{WHC (\%)=}\frac{SWM\;-DWM}{MV}\;\times 100$$

Eq (4) $$\text{TPS(\%)=}\left(1-\frac{BD}{PD}\right)\times 100$$

Eq (5) AFP(%)=TPS−WHC

### 2.4 Microgreens mineral and nitrate analysis

For mineral analysis, oven-dried ground samples were submitted to the Penn State Agricultural Analytical Services Laboratory. Samples were tested for total nitrogen using a CN autoanalyzer, following procedures reported by Vecchia et al. (2020), as well as for macro-, meso- (P, K, Ca, Mg, S, and Na) and micro (Mn, Fe, Cu, B, and Zn) minerals following acid digestion by ICP-MS standard technique (Huang and Schulte, 1985).

The salicylic acid method developed by Cataldo et al. (1975) with modification was used to analyze nitrate in microgreens shoots. Freeze-dried ground plant samples of 0.01 g were mixed with 1 mL of deionized water to extract nitrate. The mixture was vortexed for 10 s and then placed in a water bath at 45 °C for an hour. After centrifuging the samples at 5600 g for 15 min, the supernatant was used for analysis. For nitrate analysis, a mixture of salicylic and sulfuric acid (5% w/v) was combined with 20 µL of sample, standard or deionized water (for blank), while only sulfuric acid (100%) was used for the blank. After 20 min, 1.9 mL of NaOH (2 M) was added and allowed to sit for 2 min. A 300 µL portion of the mixed samples was transferred to a microplate and the absorbance was read at 410 nm using a microplate reader (Synergy H1, BioTek, Winooski, VT). Nitrate concentration was expressed on a fresh weight basis (mg nitrate/kg FW), and KNO_3_ was used as standard.

### 2.5 Phytochemical analysis

#### 2.5.1 Total chlorophyll and carotenoids

Total chlorophyll and carotenoid content of the samples were determined following the method described by Lee et al. (2021). The concentration of chlorophyll a, chlorophyll b, chlorophyll a+b, and carotenoids were expressed on a dry weight basis in mg/g DW.

#### 2.5.2 Total phenols and antioxidant activity

The concentration of total phenolic compounds in microgreens samples was determined using a modified Folin-Ciocalteu method (Ainsworth and Gillespie, 2007; Poudel et al., 2023). A 0.04 g portion of the pulverized, freeze-dried microgreens was subjected to extraction with 4 mL of 80% methanol using a sonicator (Branson CPX2800H, Branson Ultrasonics, Brookfield, CT) for 20 min, followed by 20 s of vortexing. An aliquot (1.5 mL) of the extract was transferred to a microcentrifuge tube and stored in a refrigerator (4°C) overnight in a dark environment. After 12 h, the extract was centrifuged at 1,000 rpm for two min. A total of 135 µL of distilled water, 750 µL of Folin-Ciocalteu reagent, 50 µL of the supernatant (from sample extract), and 600 µL of Na_2_CO_3_ were added to another microcentrifuge tube and vortexed for 10 s. The mixture was then incubated for 20 min at 45°C in a water bath, cooled to room temperature, and the absorbance was measured using a microplate reader at 765 nm (Synergy H1, BioTek, Winooski, VT). Total phenols content was quantified using gallic acid standards at seven different concentration levels with three replicates in 80% acetone. Total phenol concentration was expressed as gallic acid equivalent (GAE) (mg GAE/g DW) based on the dry weight.

Total antioxidant activity was determined using a modified version of the 2,2-Diphenyl-1-picrylhydrazyl (DPPH) antioxidant test reported by Herald et al. (2012) and Alrifai et al. (2020) with slight modification (Poudel et al., 2023). The sample extraction procedure was the same as described for total phenols, with a 1.5 mL aliquot stored overnight at -20°C and centrifuged at 1,000 rpm for two min the following day. A 350 mM DPPH solution was prepared in 80% methanol, and 200 µL of the solution was added to each sample, excluding the blank, which received 225 µL of 80% methanol. A 25 µL sample or standard was added to each well, and the microplate was incubated at room temperature in the dark for 6 h under a parafilm lid. Absorbance was measured at 517 nm after 6 h using a microplate reader (Synergy H1, BioTek, Winooski, VT). The antioxidant activity of the sample was expressed as Trolox equivalent (mM TEAC/g DW) using Trolox (6-Hydroxy-2,5,7,8-tetramethylchroman-2-carboxylic acid) as a standard.

### 2.6 Data analysis

All collected data except for the chemical properties of the GM were analyzed using the analysis of variance in the general linear mixed model for a split-plot design using “lmer” function in R (The R Project for Statistical Computing, Vienna, Austria). Only two subsamples of each GM were analyzed for chemical parameters, and in this case, results were presented as mean values ± standard error. All other data were subjected to the model assumption before statistical analysis. Significant means among treatments were separated using Tukey’s means separation procedure at the alpha level of 0.05. Principal component analysis (PCA) was also performed in R using the “prcomp” command to summarize the variance observed in the microgreen’s dataset.

## 3 Results

### 3.1 Growing media physicochemical chemical properties and mineral content

A comprehensive overview of the physical and chemical characteristics and mineral composition of the eight GM examined in the study (PP, CC, SMC, CMP, PP+SMC, PP+CMP, CC+SMC, CC+CMP) is presented in **Tables 1**, **2**, respectively. Organic waste compost (CMP) had the highest bulk density, followed by CC+CMP and PP+CMP, while CC and PP had the lowest bulk density (**Table 1**). Both CMP and SMC had a greater particle density than CC, PP+SMC, and PP, which had a lower particle density. The water-holding capacity of PP and CC growing media was greater than that of CMP growing medium. Total pore space was found to be greater in CC and PP growing media, however, SMC and CC+SMC had higher AFP, while PP had lower AFP.

**Table 1:** Physical properties of the alternative growing substrates tested.^1^.

Physical properties	Growing media							
	PP	CC	SMC	CMP	PP+SMC	PP+CMP	CC+SMC	CC+CMP
Bulk density (g/cm^3^)	0.13 f	0.10 g	0.22 d	0.41 a	0.17 e	0.28 c	0.15 f	0.29 b
Particle density (g/cm^3^)	1.14 b	1.03 b	1.46 a	1.51 a	1.02 b	1.30 ab	1.17 ab	1.33 ab
Water holding capacity (%)	82.83 a	81.87 a	72.00 b	65.04 c	75.70 b	72.27 b	75.41 b	71.41 b
Total pore space (%)	88.20 ab	89.95 a	83.71 bc	73.13 d	83.75 bc	78.90 cd	87.06 ab	78.07 cd
Air filled porosity (%)	5.31 c	8.06 b	11.68 a	8.03 b	7.98 b	6.59 bc	11.64 a	6.64 bc

^1^Reported values are the average of three subsamples. Means followed by different letters within each rows are significantly different at α = 0.05 via Tukey’s means separation procedure. PP: Peat-perlite medium, CC: Coconut coir, SMC: Spent mushroom compost, CMP: Compost, PP+SMC: Peat-Perlite + Spent mushroom compost (50:50, v:v), PP+CMP: Peat-Perlite + Compost (50:50, v:v), CC+SMC: Coconut coir + Spent mushroom compost (50:50, v:v), CC+CMP: Coconut coir + Compost (50:50, v:v).

**Table 2:** Chemical properties and mineral content in the growing substrate medium.^1^.

Chemical parameters	Growing media							
	PP	CC	SMC	CMP	PP+SMC	PP+CMP	CC+SMC	CC+CMP
pH	5.96 ± 0.06	6.15 ± 0.11	7.75 ± 0.23	7.80 ± 0.04	7.46 ± 0.06	7.42 ± 0.06	7.69 ± 0.01	7.66 ± 0.18
EC (mS/cm)	1.77 ± 0.01	1.33 ± 0.05	23.50 ± 1.00	11.68 ± 0.15	11.30 ± 0.30	6.13 ± 0.98	8.89 ± 0.09	5.02 ± 0.06
OM (% dry wt. basis)	56 ± 0.54	83.46 ± 0.40	64.80 ± 0.16	42.99 ± 1.14	61.94 ± 0.18	43.44 ± 0.31	70.96 ± 0.32	50.31 ± 2.02
NO_3_-N (mg/L)	80.31 ± 2.00	0.14 ± 0.10	2.88 ± 0.21	625.22 ± 3.96	44.70 ± 13.32	337.90 ± 74.96	0.09 ± 0.08	192.48 ± 6.93
NH_4_-N (mg/L)	1.95 ± 1.61	0.19 ± 0.00	39.95 ± 4.30	5.30 ± 0.20	12.90 ± 3.55	4.83 ± 0.03	15.78 ± 2.70	4.8 ± 0.00
P (mg/L)	21.58 ± 1.14	9.18 ± 0.05	62.58 ± 5.46	4.75 ± 0.19	40.02 ± 0.76	12.82 ± 2.14	25.15 ± 1.25	13.03 ± 0.04
K (mg/L)	79.80 ± 0.34	249.18 ± 6.89	6454.12 ± 678.13	1523.70 ± 7.06	2684.18 ± 16.86	781.13 ± 143.26	2088.72 ± 18.84	773.85 ± 5.95
Ca (mg/L)	147.69 ± 0.40	35 ± 0.11	1008 ± 107.31	503 ± 5.88	558.32 ± 19.79	357 ± 22.81	419 ± 8.49	419 ± 3.26
Mg (mg/L)	144.21 ± 1.52	21.40 ± .71	333.59 ± 58.20	146.37 ± 1.96	235.44 ± 12.13	135.27 ± 0.75	118.22 ± 0.39	63.01 ± 0.92
B (mg/L)	0.08 ± 0.00	0.28 ± 0.00	0.80 ± 0.05	0.73 ± 0.01	0.5 ± 0.01	0.35 ± 0.02	0.42 ± 0.00	0.27 ± 0.00
Cu (mg/L)	0.47 ± 0.29	0.20 ± 0.01	0.90 ± 0.12	0.33 ± 0.02	0.56 ± 0.02	0.23 ± 0.01	0.37 ± 0.01	0.19 ± 0.01
Fe (mg/L)	10.24 ± 0.04	6.01 ± 0.08	13.07 ± 1.89	19.66 ± 1.73	8.77 ± 0.54	16.15 ± 0.97	2.11 ± 0.07	9.05 ± 0.26
Mn (mg/L)	2.98 ± 0.21	2.06 ± 0.07	15.09 ± 1.86	4.32 ± 0.16	9.14 ± 0.12	4.29 ± 0.89	6.82 ± 0.09	6.32 ± 0.31
Na (mg/L)	42.81 ± 1.29	71.06 ± 0.38	404.02 ± 39.56	674.87 ± 6.82	206.95 ± 1.42	352.95 ± 62.87	199.07 ± 0.77	316.68 ± 5.31
Zn (mg/L)	0.91 ± 0.02	0.70 ± 0.04	10.10 ± 1.25	8.45 ± 0.35	5.87 ± 0.12	4.38 ± 0.26	3.80 ± 0.03	3.88 ± 0.05
Sulfate (mg/L)	462.42 ± 6.99	23.22 ± 1.10	7831.37 ± 823.61	488.80 ± 14.62	3869.28 ± 50.80	555.11 ± 34.05	2553.47 ± 31.04	232.23 ± 2.85

^1^Reported values are the average of two subsamples. ± are standard error. PP: Peat-perlite medium, CC: Coconut coir, SMC: Spent mushroom compost, CMP: Compost, PP+SMC: Peat-Perlite + Spent mushroom compost (50:50, v:v), PP+CMP: Peat-Perlite + Compost (50:50, v:v), CC+SMC: Coconut coir + Spent mushroom compost (50:50, v:v), CC+CMP: Coconut coir + Compost (50:50, v:v).

Growing media pH ranged between a maximum average of 7.80 ± 0.04 in CMP and a minimum average of 5.96 ± 0.06 in PP (**Table 2**). Except for PP and CC, all the other substrates tested had a pH above 7. Spent mushroom compost exhibited the highest concentration of EC, followed by CMP, PP+SMC, CC+SMC, PP+CMP, and CC+CMP, whereas PP and CC had the lowest EC concentrations, on average 7.5% and 5.6% of the EC content observed in SMC, respectively. Additionally, CC had the highest concentration of organic matter, followed by CC+SMC and SMC, while CMP and PP+CMP had lower organic matter content than other growth media tested in the study.

Examining mineral N, the highest nitrate-N concentration was found in CMP growth medium, with 85% more nitrate-N than the growth medium with the second highest concentration, PP+CMP. Conversely, the highest concentration of ammonium-N level was detected in SMC, followed by CC+SMC, and PP+SMC, respectively. Except for the content of nitrate-N, Na, and Fe, SMC had the highest content of all the other minerals. The lowest concentrations of P were observed in CMP and CC. Peat and perlite growth medium had the lowest levels of K and B, while CC growth medium exhibited the lowest levels of Ca, Mg, Mn, Zn, and sulfate.

On the other hand, the highest concentration of Fe was recorded in CMP growth medium, followed by PP+CMP and SMC. Spent mushroom compost also had the highest Zn concentration, followed by CMP and PP+SMC (**Table 2**). The samples of CMP and SMC also had high levels of Na compared to CC and PP. All the growing mixes examined had intermediate mineral content and chemical properties compared to the pure components used in each growing mix.

### 3.2 Growing media effects on microgreens yield components.

Poor germination was observed for pea and radish seeded on SMC (100%), therefore, microgreens were not harvested from trays filled with 100% SMC growing medium. An interaction effect was observed between GM and species on mean shoot fresh weight (mg/shoot), fresh yield (kg/m^2^), dry biomass (g/m^2^), and dry matter (%) content (**Table 3**). Pea microgreens had higher mean fresh weight and fresh yield than radishes, apart from PP+SMC growing medium, which provided similar mean shoot fresh weight for both species. The inclusion of SMC in the GM (PP+SMC and CC+SMC) decreased mean shoot fresh weight and fresh yield for both pea and radish microgreens. Pea microgreens grown in PP and PP+CMP accumulated higher dry biomass, while those grown in PP+SMC generated the lowest dry biomass. The dry matter content was higher in peas than in radishes and in GM containing SMC (PP+SMC and CC+SMC) for both species.

**Table 3:** Effect of growing medium on mean shoot fresh weight (mg/shoot), fresh yield (g/m^2^), dry biomass (g/m^2^), and dry matter content (%) of pea and radish microgreens.^1^.

Species	Media	Mean fresh shoot weight (mg/shoot)	Fresh yield (kg/m^2^)	Dry biomass (g/m^2^)	Dry matter (%)
Peas	PP	522.50 a	4.37 a	398.94 a	9.13 d
	CC	351.67 c	2.94 cd	293.00 b	9.97 cd
	CMP	353.61 c	2.92 cd	300.78 b	10.31 c
	PP+SMC	170.55 e	0.99 f	133.00 de	13.42 a
	PP+CMP	433.05 b	3.72 b	354.99 a	9.54 cd
	CC+SMC	255.55 d	1.72 e	200.50 c	11.66 b
	CC+CMP	372.22 bc	2.79 cd	285.20 b	10.25 c
Radish	PP	210.83 de	2.66 d	150.44 cde	5.65 f
	CC	181.39 e	2.78 cd	155.57 cde	5.59 f
	CMP	217.78 de	2.78 cd	164.40 cd	5.92 f
	PP+SMC	179.45 e	1.51 ef	106.80 e	7.16 e
	PP+CMP	219.72 de	3.72 bc	173.68 cd	5.31 f
	CC+SMC	179.45 e	1.51 ef	106.80 e	7.16 e
	CC+CMP	246.67 d	3.35 bcd	181.48 cd	5.43 f
Source of variation	Species	***	ns	***	***
	Media	***	***	***	***
	Species×Media	***	***	***	***

^1^ Reported values are averages of three replications. Significance: ns=not significant, *** P ≤0.001, respectively. Means followed by different letters within each column are significantly different at α = 0.05 via Tukey’s means separation procedure. PP: Pear-perlite medium, CC: Coconut coir, SMC: Spent mushroom compost, CMP: Compost, PP+SMC: Peat-Perlite + Spent mushroom compost (50:50, v:v), PP+CMP: Peat-Perlie + Compost (50:50, v:v), CC+SMC: Coconut coir + Spent mushroom compost (50:50, v:v), CC+ CMP: Coconut coir + Compost (50:50, v:v).

### 3.3 Growing media effects on microgreens mineral content

#### 3.3.1 Macro- and meso-minerals

Significant interaction effects were observed between species and GM on the N, P, K, Ca, Mg, S and Na content of peas and radish microgreens grown with alternative substrates (**Table 4**). The results indicated that the concentration of N was higher in pea microgreens than in radish microgreens, while the opposite was observed for the concentration of nitrate, K, Ca, Mg, and S. The highest concentration of N was found in pea microgreens grown in PP+CMP, while the highest N concentration in radish microgreens was observed in PP and CMP. The concentration of N was relatively lower in peas grown in CC or CC+SMC, and highest when CMP or PP by itself were used (PP+CMP, CMP, PP, CC+CMP), and a similar trend was observed in radish, except that the PP+SMC mix provided the lowest level of N in the case of radish. Radish accumulated more nitrate than peas when grown in PP, CMP, PP+CMP, and CC+CMP, while similar nitrate content was observed in both peas and radish microgreens when grown in CC, PP+SMC, and CC+SMC. In both microgreens, nitrate content was higher when grown in CMP followed by PP+CMP and CC+CMP and lower nitrate content was seen with CC and CC+SMC growing media. The concentration of P in radish was higher than in pea microgreens, except for microgreens grown in CMP, PP+SMC, and CC+CMP. The highest P, Ca, and Mg concentrations were observed in microgreens grown in PP media for both species. Instead, the highest K content was observed in radish microgreens grown with CC+SMC and CC+CMP media, while in peas similar concentrations of K were observed when using CMP, PP+CMP, CC+SMC, and CC+CMP. The highest S content in radish microgreens was found in PP+SMC, followed by CC+SMC and PP, while the highest S content in pea microgreens was observed in CC+SMC and PP, and the lowest concentration was observed in pea microgreens grown in CC media. Radish had a higher Na concentration than peas when grown in the same GM. The highest Na concentration was found in radish microgreens grown in CMP and CC+CMP, followed by those grown in PP and PP+CMP, and was the lowest in radish grown with CC. Similarly in the case of pea microgreens, Na concentration was the highest when using CMP and CC+CMP, followed by PP+SMC and CC, respectively, but it was the lowest in PP+SMC, PP, and CC+SMC (**Table 4**).

**Table 4:** Effects of growing media on the macro- and meso-mineral profile of pea and radish microgreens.^1^.

Species	Media	Nitrate	N	P	K	Ca	Mg	S	Na
		mg/kg FW	%						
Peas	PP	233.43 ef	8.05 abc	0.83 c	2.77 g	0.42 d	0.43 cd	0.71 fg	0.06 h
	CC	152.33 f	7.54 d	0.69 gh	3.15 fg	0.15 g	0.24 fg	0.38 k	0.09 gh
	CMP	623.11 c	8.28 ab	0.72 fgh	4.06 e	0.32 e	0.21 fg	0.47 jk	0.28 e
	PP+SMC	204.64 f	7.99 bc	0.66 h	3.31 f	0.17 g	0.21 g	0.63 gh	0.05 h
	PP+CMP	433.09 d	8.35 a	0.77 def	4.06 e	0.30 e	0.25 f	0.59 hi	0.17 fg
	CC+SMC	215.69 f	7.78 cd	0.71 fgh	4.24 e	0.21 fg	0.21 g	0.78 f	0.06 h
	CC+CMP	405.86 de	8.30 ab	0.76 def	4.00 e	0.26 ef	0.22 fg	0.49 ij	0.23 ef
Radish	PP	563.06 cd	7.05 e	1.09 a	4.34 e	0.96 a	0.79 a	1.96 c	0.79 b
	CC	144.43 f	4.57 h	0.95 b	6.36 d	0.57 c	0.47 bc	1.67 de	0.55 d
	CMP	1593.17 a	6.90 e	0.75 efg	7.47 bc	0.97 a	0.42 d	1.58 de	0.95 a
	PP+SMC	163.54 f	5.40 g	0.72 fgh	7.14 c	0.66 bc	0.46 bcd	2.30 a	0.26 ef
	PP+CMP	1053.83 b	6.48 f	0.84 c	7.44 bc	0.89 a	0.48 b	1.68 d	0.69 c
	CC+SMC	164.49 f	4.63 h	0.82 cd	8.05 a	0.69 b	0.38 e	2.18 b	0.31 e
	CC+CMP	911.36 b	6.53 f	0.81 cde	7.94 ab	0.92 a	0.43 d	1.57 e	0.88 a
Source of variation	Species	***	***	***	***	***	***	***	***
	Media	***	***	***	***	***	***	***	***
	Species×Media	***	***	***	***	***	***	***	***

^1^Reported values are averages of three replications. Significance: *** P ≤0.001. Means followed by different letters within each column are significantly different at α = 0.05 via Tukey’s means separation procedure. PP: Pear-perlite medium, CC: Coconut coir, SMC: Spent mushroom compost, CMP: Compost, PP+SMC: Peat-Perlite + Spent mushroom compost (50:50, v:v), PP+CMP: Peat-Perlie + Compost (50:50, v:v), CC+SMC: Coconut coir + Spent mushroom compost (50:50, v:v), CC+ CMP: Coconut coir + Compost (50:50, v:v).

#### 3.3.2 Microminerals

A significant interaction effect between species and GM was seen only in the case of Zn, B and Cu; Mn concentration was affected by both species and GM, while only the species influenced the concentration of Fe (**Table 5**). The concentration of Zn was relatively higher in peas compared to radish, but it was highest in peas grown on CC+SMC and PP, while in the case of radish it was highest when using PP and CC. The concentration of Mn was on average higher in radish microgreens compared to peas, and across species it was affected by GM used, with CC and PP providing higher Mn concentration compared to other media tested and CMP providing the lowest concentration of Mn in both species. The concentration of B ranged between 36.67 and 13.00 mg/kg in radish microgreens grown in CC and PP+SMC, respectively, and it ranged between 20.67 and 12.00 mg/kg in pea microgreens grown on CC and PP+SMC, respectively; however, different trends were observed for the two species grown on other GM tested. Cu concentration was on average higher in pea microgreens than in radish, and while it was not affected by GM in the case of pea microgreens, it was higher in radish microgreens grown in PP compared to radish grown on other substrates.

**Table 5:** Effects of growing media on the micro-mineral profile of pea and radish microgreens.^1^.

Species		Fe	Zn	Mn	B	Cu
	Media	mg/kg DW				
Peas	PP	81.67	71.33 a	29.00	15.33 fg	11.67 ab
	CC	82.00	67.33 ab	28.33	20.67 d	13.00 a
	CMP	72.33	63.67 b	19.33	18.33 de	12.00 a
	PP+SMC	74.67	64.67 b	18.00	12.00 h	13.00 a
	PP+CMP	88.00	64.67 b	24.33	17.33 ef	11.33 ab
	CC+SMC	84.67	72.00 a	21.00	17.68 ef	12.33 a
	CC+CMP	85.67	66.67 ab	20.00	17.00 ef	12.33 a
Radish	PP	76.67	55.33 c	30.67	19.67 de	8.33 bc
	CC	70.68	54.67 c	33.33	36.67 a	5.00 c
	CMP	155.70	42.33 e	23.68	34.33 ab	5.00 c
	PP+SMC	91.67	47.00 de	27.00	13.00 gh	5.33 c
	PP+CMP	102.30	43.67 de	24.67	33.67 bc	5.00 c
	CC+SMC	66.67	48.33 d	26.33	18.67 de	5.00 c
	CC+CMP	167.00	42.67 e	28.33	31.00 c	5.33 c
Source of variation	Species	*	***	**	***	***
	Media	ns	***	***	***	ns
	Species×Media	ns	***	ns	***	*

^1^Reported values are averages of three replications. Significance: ns=not significant, * P ≤0.05, ** P ≤ 0.01 or *** P ≤0.001, respectively. Means followed by different letters within each column are significantly different at α = 0.05 via Tukey’s means separation procedure. PP: Pear-perlite medium, CC: Coconut coir, SMC: Spent mushroom compost, CMP: Compost, PP+SMC: Peat-Perlite + Spent mushroom compost (50:50, v:v), PP+CMP: Peat-Perlie + Compost (50:50, v:v), CC+SMC: Coconut coir + Spent mushroom compost (50:50, v:v), CC+ CMP: Coconut coir + Compost (50:50, v:v).

### 3.4 Growing media effects on microgreens phytochemicals content

#### 3.4.1 Total chlorophyll and carotenoids

Species and GM had an interaction effect on chlorophyll a, chlorophyll b, chlorophyll a+b, and carotenoids (**Table 6**). Chlorophyll a was higher in pea microgreens when grown in PP+SMC compared to peas grown in PP+CMP, PP and CMP, while no differences were observed among other GM tested. Instead, in radish microgreens, chlorophyll a was higher in PP+SMC and CC+SMC than in PP, but similar when grown using other media. Chlorophyll b and a+b were lower in radish microgreens grown in PP compared to other GM, and a similar effect was observed for the carotenoid content. In the case of pea microgreens, chlorophyll b, and chlorophyll a+b were higher when using CC and PP+SMC compared to PP+SMC but were similar when using other GM. Carotenoid concentration in pea microgreens was higher in CC compared to PP+CMP and CC+CMP but was similar when using other GM. Overall, in pea microgreens a higher photosynthetic pigment content was observed in CC and GM mixes containing SMC (PP+SMC and CC+SMC).

**Table 6:** Effect of the species and growing media on chlorophyll a, chlorophyll b, chlorophyll a+ b, and carotenoid content.^1^.

Species	Media	Chlorophyll a	Chlorophyll b	Chlorophyll a+ b	Carotenoids
		mg/g DW			
**Peas**	PP	0.83 bcd	0.48 ab	1.24 abc	0.67 abc
	CC	1.09 ab	0.60 a	1.60 a	0.76 a
	CMP	0.85 bcd	0.49 ab	1.28 abc	0.67 abcd
	PP+SMC	1.14 a	0.54 a	1.59 a	0.57 abcde
	PP+CMP	0.65 de	0.37 bc	0.96 cd	0.45 ef
	CC+SMC	1.03 ab	0.49 ab	1.47 ab	0.70 ab
	CC+CMP	0.86 abcd	0.44 abc	1.23 abc	0.50 bcdef
**Radish**	PP	0.42 e	0.27 c	0.66 d	0.30 f
	CC	0.81 bcd	0.46 ab	1.21 abc	0.43 ef
	CMP	0.73 cd	0.48 ab	1.16 abc	0.45 cdef
	PP+SMC	0.95 abc	0.49 ab	1.36 abc	0.43 ef
	PP+CMP	0.70 cd	0.45 ab	1.11 bc	0.45 cdef
	CC+SMC	0.90 abcd	0.48 ab	1.31 abcg	0.42 ef
	CC+CMP	0.66 de	0.46 ab	1.07 bcd	0.44 def
**Source of variation**	Species	**	ns	*	**
	Media	***	***	***	*
	Species×Media	*	**	**	***

^1^Reported values are averages of three replications. Significance: ns=not significant, * P ≤0.05, ** P ≤ 0.01 or *** P ≤0.001, respectively. Means followed by different letters within each column are significantly different at α = 0.05 via Tukey’s means separation procedure. PP: Pear-perlite medium, CC: Coconut coir, SMC: Spent mushroom compost, CMP: Compost, PP+SMC: Peat-Perlite + Spent mushroom compost (50:50, v:v), PP+CMP: Peat-Perlie + Compost (50:50, v:v), CC+SMC: Coconut coir + Spent mushroom compost (50:50, v:v), CC+ CMP: Coconut coir + Compost (50:50, v:v).

#### 3.4.2 Total phenols and antioxidants

**Figure 1:**
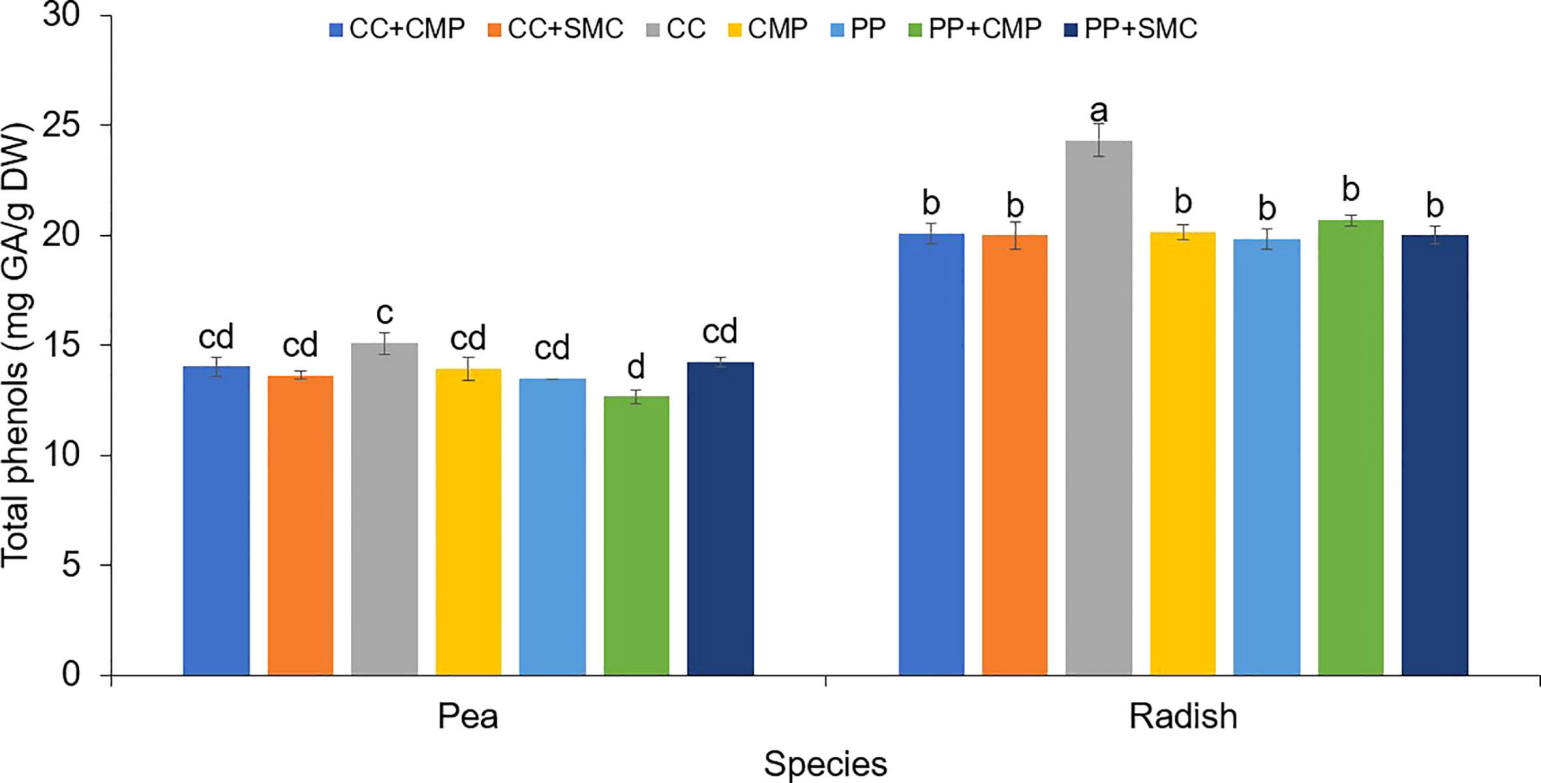
Growing media and species interaction effect on the total phenols (mg GA/g DW) in pea and radish microgreens. Vertical bars indicate average values and the standard error. Different letters indicate significant differences at P = 0.05 *via* Tukey’s means separation procedure. PP, Pear-perlite medium; CC, Coconut coir; SMC, Spent mushroom compost; CMP, Compost; PP+SMC, Peat-Perlite + Spent mushroom compost (50,50; v,v); PP+CMP, Peat-Perlie + Compost (50,50; v,v); CC+SMC, Coconut coir + Spent mushroom compost (50,50; v,v); CC+ CMP, Coconut coir + Compost (50,50; v,v).

**Figure 2:**
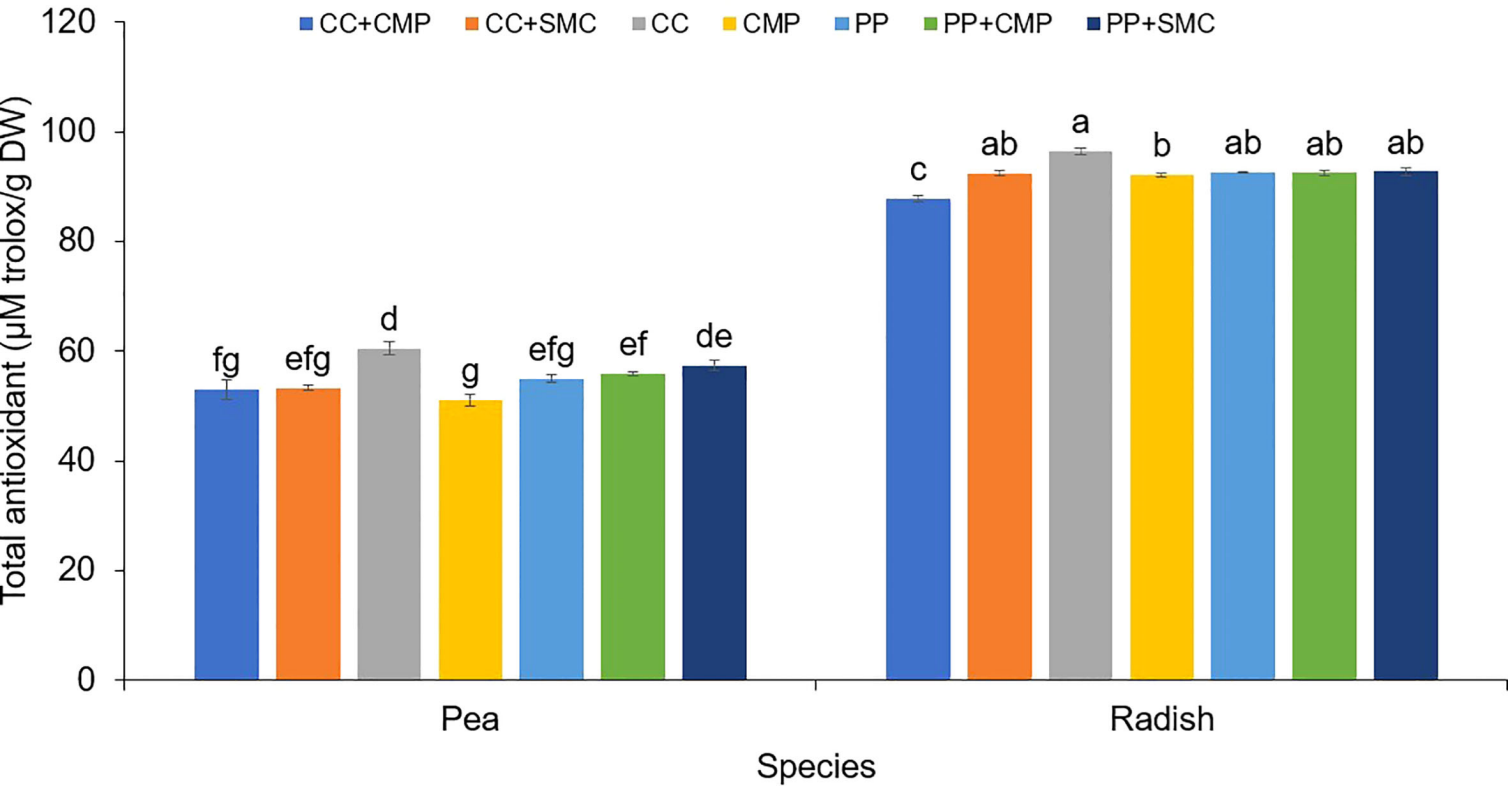
Growing media and species interaction effect on the total antioxidant activity (mg Trolox/g DW) in pea and radish microgreens. Vertical bars indicate average values and the standard error. Different letters indicate significant differences at P = 0.05 *via* Tukey’s means separation procedure. PP, Pear-perlite medium; CC, Coconut coir; SMC, Spent mushroom compost; CMP, Compost; PP+SMC, Peat-Perlite + Spent mushroom compost (50,50; v,v); PP+CMP, Peat-Perlie + Compost (50,50; v,v); CC+SMC, Coconut coir + Spent mushroom compost (50,50; v,v); CC+ CMP, Coconut coir + Compost (50,50; v,v).

An interaction effect between species and GM was observed on microgreens’ total phenols (**Figure 1**) and antioxidant activity (**Figure 2**). On average radish microgreens had higher total phenols and antioxidant activity compared to peas, however, the GM effect was slightly different between the two species. Pea microgreens had higher total phenols content when grown in CC compared to PP+CMP, but no differences were observed among other GM. While radish microgreens had higher total phenols when grown in CC media compared to all the other GM tested.

In both pea and radish microgreens, total antioxidant activity was the highest when using CC. However, while in pea microgreens, the lowest antioxidant activity was observed when using CMP, in the case of radish microgreens, total antioxidant activity was higher in CC only when compared to radish grown in CC+CMP and CMP, but it was similar among other GM tested (**Figure 2**).

### 3.5 Principal component analysis

**Figure 3:**
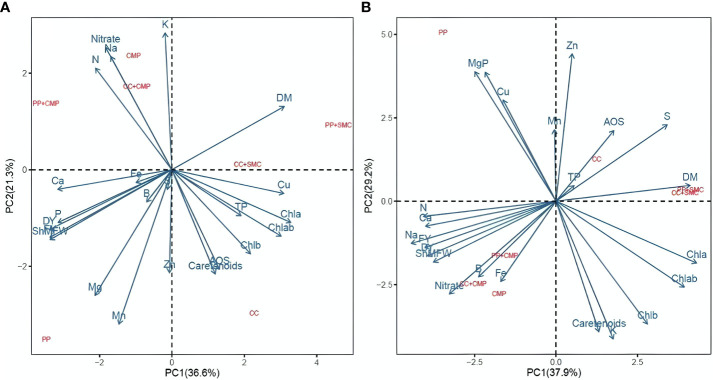
Principal component analysis biplot (PC1 vs. PC2) showing the spatial distribution of the yield parameters, mineral profile, and nutritional quality parameters of peas **(A)** and radish **(B)** microgreens grown in different growing media. FY, Fresh yield; DY, Dry yield; DM, Dry matter; ShMFW, Shoot mean fresh weight; TP, Total phenol; AOD, Antioxidant activity; Chla, Chlorophyll a; Chlb, Chlorophyll b; Chlab, Chlorophyll a + b; PP, Pear-perlite medium; CC, Coconut coir; SMC, Spent mushroom compost; CMP, Compost; PP+SMC, Peat-Perlite + Spent mushroom compost (50,50; v,v); PP+CMP, Peat-Perlie + Compost (50,50; v,v); CC+SMC, Coconut coir + Spent mushroom compost (50,50; v,v); CC+ CMP, Coconut coir + Compost (50,50; v,v).

Principal component analysis (PCA) included all the variables measured on microgreens to summarize the results presented in **Tables 2**–**5** and **Figures 1** , **2**. The PCA was conducted separately for each species to better understand the GM’s effect on the microgreens’ yield, mineral profile, and nutritional quality, as there were interactions between the species and the GM in most of the variables studied. **Figure 3** shows the biplot of the PCA analysis conducted on the yield, mineral, and nutritional profile of the peas (3A) and radish (3B) microgreens. The PCA plots revealed varied effects of the GM based on the species tested. In pea microgreens, PC1 explained 36.6% of the variance in the data, while PC2 accounted for 21.3%. The PP medium was associated with fresh yield, dry yield, Mg, B, Mn, P, and Ca content in pea microgreens, while CC media was associated with photosynthetic pigments, carotenoids, total phenols, antioxidant activity, and Cu (**Figure 3A**). Additionally, the dry matter content of pea microgreens was related to GM containing SMC (CC+SMC and PP+SMC), while nitrate, N, K and Na content was associated with media containing CMP (CMP, PP+CMP, and CC+CMP).

In the case of radish microgreens, PC1 explained 37.9% of the total variance of the data, while PC2 covered 29.2% of the total variance distribution (**Figure 3B**). **Figure 3B** also revealed a clear association of certain variables studied with the GM used to cultivate radish microgreens. The use of PP mix was associated with Mg, P, Cu, and Mn content in radish microgreens, while fresh yield, dry yield, shoot mean fresh weight, and Fe, B, Al, Na, Ca, nitrate and N content in radish microgreens were linked with media containing CMP (CMP, PP+CMP, and CC+CMP). Like for pea microgreens, the nutritional parameters, such as total phenols and antioxidant activity of radish microgreens, were positively associated with CC growing media. Additionally, the dry matter content was related to GM containing SMC (CC+SMC and PP+SMC). For both species the PCA evidenced clear separation between PP, CC, and substrates containing CMP and SMC. At the same time, substrate mixes containing CMP and SMC were grouped in the same quadrant of the biplot (**Figure 3**).

## 4 Discussion

The results of the present study showed a wide variation in the physical properties of tested GM. All media had a desirable BD (<0.4 g/cm^3^), as indicated by Abad et al. (2001), except for CMP, which had a BD of 0.41 g/cm^3^ (**Table 1**). Bulk density is an indicator of the substrate compaction and is inversely correlated to GM’s porosity, as CMP media also had the lowest total pore space (73.13%) and water-holding capacity (65.04%). Low bulk density is desirable as high BD indicates a higher transportation cost and low porosity (Di Gioia et al., 2017a). The particle density of tested GM was 1.03-1.51 g/cm^3^, where CC had the lowest and CMP and the highest PD. Water holding capacity (WHC) was lower in CMP, followed by media containing SMC alone or mixes of CMP and SMC. Only the CC, PP, and CC+SMC had ideal pore spaces greater than 85% (Abad et al., 2001). Higher pore space and air-filled porosity indicate the ideal condition for promoting gas exchange by the root system (Di Gioia et al., 2017a). Higher air-filled porosity was measured in the SMC and CC+SMC, while PP had the lowest air-filled porosity. Higher air-filled porosity is generally associated to lower WHC suggesting that more frequent irrigation with smaller volumes are required to ensure an optimal water status of the crop (Di Gioia et al., 2017a).

A large variation of the chemical properties of the GM tested was observed as well (**Table 2**). The ideal GM should have a pH range of 5.5-6.5 and low electrical conductivity,<0.5 mS/cm (Kyriacou et al., 2016; Di Gioia et al., 2017a). Only PP and CC had a pH within the optimal range for GM, while all other GM tested had a pH higher than 6.5 (**Table 2**). None of the media tested had an EC level below 0.5 mS/cm; however, PP and CC had much lower EC than SMC (23.50 mS/cm) and CMP (11.68 mS/cm). The high levels of salinity observed in SMC were not consistent with those reported by Fidanza et al. (2010) and resulted in very poor germination of both radish and pea microgreens, suggesting that such level of salinity inhibited seed germination and was phytotoxic (Barker et al., 1970). Based on these results it was evident that SMC by itself is not a suitable growing medium to produce microgreens. The high level of salinity observed in SMC was reflected in the high concentration of ammonium-N, P, K, Ca, Mg, B, Cu, Mn, Zn, and sulfate ions. Besides the high EC level, another factor that could potentially explain the poor germination and phytotoxic effect of SMC is the high content of NH_4_-N and the consequent low molar NO_3_:NH_4_ ratio that characterized this substrate, which may have negative effects on seed germination and plant growth (Barker et al., 1970; Petropoulos et al., 2019a). Mixing SMC (50:50, v:v) with PP and CC (PP+SMC and CC+SMC) resulted in substrate mixes characterized by a lower pH and substantially decreased the EC and the concentration of most ions. When used in a mix with PP and CC, SMC did not negatively affect the germination of the two microgreen species. However, SMC negatively affected fresh and dry yield and the average single shoot fresh weight, suggesting that such levels of EC and ions were still relatively high, and that SMC should probably be used in PP- and CC-based media mixes in proportions that are below 50%. A high EC level was also observed in CMP; however, it did not negatively affect the germination of peas and radish microgreens. Mixes of CMP with PP and CC further reduced the EC and pH level and those substrate mixes had higher NO_3_-N: NH_4_-N ratio, Fe, Ca, and K compared to other substrate mixes, which possibly contributed to enhance microgreen yield.

Pea microgreens had higher mean fresh weight and fresh yield than radishes, apart from the PP+SMC growing medium, which provided similar mean shoot fresh weight for both species. The inclusion of SMC in GM (PP+SMC and CC+SMC) decreased mean shoot fresh weight and fresh yield for both pea and radish microgreens. Pea microgreens grown in PP and PP+CMP accumulated higher dry biomass, while those grown in PP+SMC generated the lowest dry biomass. The dry matter content was higher in peas than in radishes and GM containing SMC (PP+SMC and CC+SMC) for both species. These results are consistent with the findings of previous studies indicating that an increase of the EC or salinity can limit plant water uptake, thereby reducing plant growth and increasing plant tissue dry matter content (Di Gioia et al., 2018b; Islam et al., 2019).

The observed interactive effects between species and GM on the microgreen yield components suggest that the two species have slightly different needs in terms of growing substrates. Except for PP+SMC, pea microgreens took more advantage of PP-based media (PP and PP+CMP), resulting in relatively higher fresh and dry yield and single shoot mean fresh weight compared to other substrates tested. Instead, radish microgreens thrived more in CMP-based mixes (PP+CMP, CC+CMP), suggesting that radishes took more advantage of the relatively higher levels of nutrients such as NO_3_-N, K, and Ca available through CMP-based mixes compared to PP-based substrates preferred by peas. On the other hand, the preference of pea microgreens for PP-based media (PP and PP+CMP) could be explained by the appreciation of the species for a relatively lower pH and EC level. The difference in yield observed between peas and radish microgreens is consistent with the results reported by Li et al. (2021) and Xiao et al. (2012) and is explained by their genetic diversity and the difference in size of the seeds of the two species. The yield decline observed in the two species grown in both substrate mixes containing SMC (PP+SMC and CC+SMC) was consistent with the high dry matter content observed in the same greens. Such results are likely due to the salinity stress caused by the high EC levels and the lower water holding capacity of the two SMC mixes (CC+SMC and PP+SMC). Several studies have shown a positive correlation or increase in DM content with salinity stress or increased EC levels (Ashbell and Evers, 2000; Ding et al., 2018; Corrado et al., 2021). Except for SMC-containing substrate mixes, all the other media tested provided microgreens with dry matter levels within the ranges considered typical for brassica and pea microgreens (Santos et al., 2014; Xiao et al., 2016; Di Gioia et al., 2017a; Di Gioia et al., 2019a).

Consistently with previous studies, besides affecting yield and its components, GM tested had considerable effects on the mineral profile of both microgreen species (Di Gioia et al., 2017a). Comparing the two species across GM, a higher K, Ca, Mg, B, Na, S, Mn, and Fe was observed in radish compared to pea microgreens, while N, Cu and Zn concentrations were higher in peas than in radish microgreens (**Table 4**, **5**). Moreover, examining the micromineral profile of the two microgreen species, some differences were observed in terms of relative abundance of different macrominerals. Peas accumulated macrominerals in the following decreasing concentration order N > K > P > S > Ca > Mg > Na, while radish accumulated macrominerals in the following decreasing concentration order K > N > S > P > Ca > Mg > Na, respectively. The relatively high content of N in peas was expected, considering that as a leguminous pea tends to have relatively high amino acid and protein content in seeds and leaves (Dovrat et al., 2020; Kowitcharoen et al., 2021) compared to brassicas. While relatively higher levels of S were expected in radishes, as brassicas biosynthesize relatively high quantities of glucosinolates, which are bioactive organosulfur compounds generally positively correlated to S content and providing the pungent mustard flavor that characterize brassicas (Di Gioia et al., 2018a; Di Gioia et al., 2019b). The argument discussed above was also supported by the PCA analysis that showed a strong correlation among total phenols, antioxidant capacity and S content in the radish microgreens (**Figure 3B**).

The trend of macro- (P, K, Ca, and Mg) and micro-mineral (Mn, Fe, Cu, B, and Zn) concentrations in both peas and radish microgreens did not reflect the levels of minerals present in the GM except for Na, NO_3_-N, and sulfate (**Table 2**, **4** , **5**). While the two species modulated the concentration of minerals, the pH and EC of the GM might have influenced the availability and uptake of macro- and micro-minerals more than the actual mineral concentration of each GM tested (Bonasia et al., 2017; Di Gioia et al., 2018b; Nerlich and Dannehl, 2021), resulting in the interaction effect observed between species and substrates. However, the high N levels observed in both pea and radish microgreens grown in media such as PP+CMP, CC+CMP, CMP, and PP aligned with the relatively high levels of NO_3_-N observed in those GM compared to others. Likewise, the higher S content observed in radishes grown with substrate mixes containing SMC (CC+SMC and PP+SMC) is consistent with the high sulfate levels present in those media (**Table 2**). These results are in agreement with the findings of several studies that have associated an increased accumulation of S and sulfur-containing glucosinolate in brassica species in the presence of increased availability of sulfur in the GM or nutrient solution (Zhou et al., 2013; Di Gioia et al., 2018b; Di Gioia et al., 2019b).

The higher photosynthetic pigments observed in peas compared to radish is consistent with the findings of other authors (Wojdyło et al., 2020) and could be associated with the higher level of N observed in peas compared to radish across alternative GM tested. Analyzing the effects of the substrates tested, higher photosynthetic pigments content was observed in microgreens grown in media mixes containing SMC (PP+SMC, CC+SMC) compared to other GM tested (**Table 6**). Such results contrast with the lower microgreens yield observed in the same treatments, and the higher EC levels observed in SMC-containing substrates compared to other media tested. While a decrease in chlorophyll content has been observed in different crops when exposed to increasing levels of salinity (Jamil et al., 2007; Giménez et al., 2021), other authors observed increased chlorophyll content in the presence of moderate salinity stress conditions (Wang et al., 2013; Islam et al., 2019). The EC levels recorded in SMC mixes could cause mild salinity stress, which could have determined a slight increase of chlorophyll content consistently with previous studies. The higher total phenols and antioxidant capacity observed in radish microgreens compared to peas is in agreement with the findings of Kowitcharoen et al. (2021) and Truzzi et al. (2021). The higher antioxidant capacity of radish microgreens could be due to the higher content of phenolic compounds and compounds like glucosinolates, which have antioxidant properties and are present in radish but not in pea microgreens (Petropoulos et al., 2017; Di Gioia and Petropoulos, 2021). The higher antioxidant capacity detected in microgreens grown on CC media, characterized by a low EC level (1.33 ds/m) compared to other GM tested, is consistent with the findings of Ren et al. (2022) and Xiong et al. (2017) who found higher total phenols and antioxidant in tomato grown in CC compared to peat-moss and rockwool and in basil plants exposed to low EC levels (0.5-1 dS/m) compared to high EC (3 and 5 dS/m) of the nutrient solution.

As indicated by the PCA biplots, the effect of the GM on yield, mineral profile, and nutritional quality was modulated by the microgreen species. The fresh yield of pea microgreens was positively associated with PP growing media, while PP+CMP was a more suitable media for radish microgreens in terms of fresh yield. The PCA pointed out that DM content was related to the media containing SMC (PP+SMC and CC+SMC) and total phenols and antioxidants with CC-growing media regardless of the species grown. Aiming to assess the potential of CMP and SMC as alternative GM and peat-substitute in the production of microgreens, the results of this preliminary study suggest that locally available CMP used in mix with PP may be a suitable substrate for the sustainable production of microgreens with minimum trade-off in terms of yield compared to the use of peat-based GM. For instance, PP+CMP resulted in a 14.74% lower yield in peas and a 28.5% higher yield in radishes compared to standard PP media while providing microgreens of similar or higher nutritional value. Although GM mix containing 50% SMC resulted in a lower fresh yield for both microgreens, the results of this preliminary study suggest that SMC mixed with PP and CC in proportions below 50% could dilute the negative effect of SMC allowing to adequately grow microgreens. While this preliminary study allowed to discern the potential of CMP and SMC as alternative growing substrate and peat-substitute to produce microgreens, further research is needed to assess the use of SMC and CMP in mix with PP and CC at various proportions and to evaluate the environmental and economic benefits of substituting peat with locally available CMP and SMC for a more sustainable production of microgreens.

## 5 Conclusion

The present research suggests that the use of locally sourced recycled organic materials, such as CMP, and their combination with standard GM such as PP and CC is a potential opportunity for enhancing the sustainability of the production of microgreens. Examining eight alternative GM for the production of pea and radish microgreens, a significant impact was observed on yield, mineral content, and nutritional value of both microgreen species, and such effects were modulated by the species. Peat and perlite provided the highest yield in the case of pea microgreens, while a mixture of PP and CMP (50:50, v/v) was optimal for radish microgreens. The results also indicated that mixing CMP with PP can produce microgreens with comparable or higher nutritional quality compared to PP, albeit with a potential trade-off in yield. Considering that CMP is locally available at low cost and is environmentally more sustainable than peat and peat-based media, its use at least as a partial peat-substitute could reduce the reliance of the microgreens industry on peat and allow a more sustainable production of microgreens. In addition, future research evaluating the commercial application of CMP and SMC as alternative growing substrate should assess the microbiological quality and safety of microgreens.

## Data availability statement

The study’s original contributions are included in the article. For further information, please contact the corresponding author.

## Author contributions

FD developed the idea. FD and PP designed and coordinated the study. PP and AD conducted the experiment and collected data. FD provided supervision and the resources to conduct the experiment. PP managed and analyzed the data. PP and FD interpreted the results. PP wrote the first draft of the manuscript. FD and AD contributed to editing the manuscript. All authors contributed to the article and approved the submitted version.
